# Chit-Chat

**Published:** 1874-04

**Authors:** 


### Editorial Chit-Chat.

A Bull.—A coroner's jury in Chicago returned a verdict of "died from premature birth."

Too Bad.—We hear the young men complaining that the ladies give no thought of those of their number who "keep away from saloons."

Another.—Our city has just had another large fire, in which $300,000 worth of property was destroyed, and that upon the principal street in the city.

Severe Struggle.—Our ministers have their hands full just now, fighting the devil and the Beecher family. Their success is not brilliant in either instance.

Fashion.—Dr. Mary Safford Blake is lecturing on dress reform, in Boston. Her audiences are described as presenting a "very fashionable appearance."

Probable.—A Quack medicine dealer sends us an advertisement for some competent person to undertake the sale of some wonderful compound, and adds, "it will prove highly lucrative to the undertaker." We do not doubt it.

Rough.—The Chicago *Times* is very severe upon Dr. H. A. Johnson, of that city, accusing him of willfully killing the wife of the editor, with a dose of morphine. Editor and Doctor are abusing each other shamefully through the papers. Will some friend send us a history of the case? We confess we cannot understand it.

Law and Medicine.—It often happens that "smart" young limbs of the law take great delight in asking superfluous questions of medical witnesses, when on the stand, with a view to puzzling or annoying them. One such was cross-examining the celebrated French Chemist Orfila, and put him the question whether he could state the precise amount of arsenic requisite to kill a fly. "Certainly," replied the expert; "but I must know beforehand the age of the fly, its sex, its temperament, its condition, and habits of body, whether married or single, widow or maiden, widower or bachelor."

Insane.—The Chicago medical journals are authority for saying that two hundred and fifty persons have been adjudged insane by reason of the Chicago fire.

Clever of Them.—H. says the Indians have just completed a new method for scalping their victims, so that a bald headed man stands no better show than any one else. This, he thinks, is as it should be.

Roller Towels.—They are a nuisance, when employed in public places, like hotels or restaurants. Never use them. All sorts of people, afflicted with all sorts of diseases, wipe their nasty faces upon them. We recently met with two cases of destructive inflammation of the eyes, traced to the patient's wiping upon one of the towels referred to. When traveling, carry your own towel, comb, brush and soap. It is much the cleanliest plan, and decidedly the safest.

Fastidious, Very.—The Cincinnati *Commercial* relates of a young physician of that city, who recently took a young lady for a drive. Passing a cemetery on the road, the doctor alighted to say a few words to the sexton, and upon returning to the young lady, surprised her by asking whether she would object to riding home with a corpse, which he had just bought. Singularly enough, the young lady declined, and has since formed a resolution not to go riding with any more enthusiastic students of medicine.

Good.—A law has long been needed to discourage "emotional insanity," that form that allows one fellow to shoot and kill another, and then escape punishment under the plea of insanity. One has been introduced into the Ohio legislature which we sincerely hope may not only become the law in that State, but in all others as well. It provides that where insanity is set up as a defense in cases of killing, the prisoner shall undergo a preliminary trial to decide upon the justice of his plea, and the exact measure of accountability. If he be adjudged sane, trial will, of course, proceed in the ordinary form, but if he be found insane he is at once committed to a lunatic asylum.—Pending his incarcertion therein the indictment still hangs over him, and if he recovers his senses he will undergo his trial.

Signs of the Times.—"Prayed out of Town," are the signs nailed over the doors of some of the liquor saloons in Ohio.

---

Delay.—Our magazine is a litte late this quarter, in consequence of the delay in receiving our paper. It will be observed that the printers have furnished us with most excellent material and skillful typography, so that the Bistoury will compare favorably with most first-class magazines.

---

Read It.—We are not in the habit of calling attention to our advertisments, as there are so few of them, they are always prominent; but you must read Mr. Dexter's full page, on the outside cover. He not only has one of the handsomest stores in the city, but everything in his line is found in it.

---

Twins.—And this time it is Kalamazoo that possesses the curiosities,—the smallest twins ever heard of,—a boy and girl, weighing together three pounds, and in good health.—Send them to the Philadelphia doctors for dissection—it would be a good advertising dodge, which they could adopt without violating the code, you know.

---

Ten Years.—We remind our readers that this is the tenth year of the publication of the Bistoury. In looking over our subscription list, we find that we have lost 64 subscribers, during this time, from death, and less than 20 through suspension of their subscription. It is strange how familiar the old names have become to us. We recognize them at once, as we add name after name upon the same page with them,—theirs seem to shine more brightly from among them. We send thanks and kindly greetings for their ten years' patience with us, and are wondering the while, how many of us may survive the ten to come. Doubtless, many will be missed from the list, and perhaps the Editor himself gathered to his fathers:—We shall see.

---

How is This?—The following is an exact copy of a petition now being circulated in Massachusetts for signatures: "We, the undersigned, citizens of Massachusetts, and consumers of tobacco, protest against any advance in the rate of tax on what we consider one of the necessaries of life and a great help in the temperance reform now going on in this State."

Railway Accidents.—We need legislation compelling Railway companies to adopt some safe apparatus for coupling cars. The accidents that are continually occurring among brakemen, by reason of the very imperfect and dangerous method of performing this work, are appaling. Railroad accidents in the United States average two persons killed and six injured every working day in the year.

---

Patent Medicine Proprietors.—We cannot agree with our contributor Mr. Bidwell, in his assertion that the proprietors of the well known nostrums to which he refers, are "regular practitioners of medicine." Some of them are not physicians at all, nor never were; but simply assumed the title of "Doctor" *because* they are dealing in so-called medicines. Those of them who really are physicians, never reached any degree of eminence in their profession, and probably their names would never have reached the public in any other manner than through the posters upon our bill-boards and fences. No intelligent or capable physician is ever found engaged in the manufacture of any secret nostrum, recommended for the cure of disease.

---

The Women.—We think their abilities have always been underestimated. We never fully appreciated their versatile capabilities until placed under the same roof and in constant communion with twenty or thirty of them.—Gather that number of ladies into one family, and you will have secured an amount of talent and ingenuity equal to any emergency that could possibly arise. You will find among their numbers, capable nurses, exquisite singers, fine readers, splendid players, with other talents and accomplishments so diversified that at any moment an entertainment could be organized of a character that could not fail of interesting almost any audience. If our readers could but see our "crusaders" on one of their evening "sprees," they would have the pleasure of witnessing a performance that would dispel the "blues," cheer up the drooping spirits of the afflicted, or drive you frantic, as occasion might require.

---

"Better Late Than Never."—The origin of this expression is not clear to us, but we have heard it—heard it repeatedly, in fact, and have been struck with its relevancy to the object now in view, viz: our desire to write a word of applause for the improved appearance of the *Daily Advertiser*, since its enlargement and adoption of its new dress. You see, all this occurred three months ago, after the last number of the Bistoury had been issued, giving every newspaper within our reach, opportunity to say pretty things of the *Advertiser*, while we could only read and admire. But we will have our say ; and having an eye to the beautiful in typography, we declare, with much satisfaction, that a better edited, printed and arranged newspaper does not come to our table.

Few readers of their morning paper appreciate the amount of labor that is performed by its editors, to present their patrons with the telegraphic and local news of the day. It requires a wonderful amount of skill, energy and mental force to collect, cull, and curtail the news into those short, crisp and newsy paragraphs, which we all recognize as from the pen of the *Advertiser's* local editor, Mr. Ausburn Towner. The only compliments we ever give him, is to occasionally throw the paper down to remark that, "the *Advertiser* is dull this morning ;" and presently our eye catches the item "our local editor is out of the city to-day." Another indefatigable worker is Capt. Dumars—'tis he who arranges the telegraphic news, giving us the startling "head lines," and condensing the news items into just the proper space for us to read, a matter requiring rare talent, and enforcing his presence far into the night.—Much might be written of the different workers upon our daily press, requiring more space than our printer is willing to afford us, so we must defer it to another time.

---

Ye Olden Time.—It *has* always been the case, and, doubtless always *will* be, that people will resort to all manner of nostrums, and consult all sorts of lunatics upon matters relating to health. Superstition has prevailed at all ages of the world and promises to continue to the end of time.

Why, even insects, those nasty little pests at which ladies are wont to scream, whenever they come in contact with them, had great prominence in the *materia medica* of the superstitious sick, in the days of Pliny. These disgusting little rascals were absolutely swallowed, by the poor sick people with full confidence in their efficacy and with as much gusto as are our pills and potions of to-day.

We read, in an old work upon the medicines of centuries ago, that five gnats were equal to three grains of calomel. A lady-bird was considered a sovereign remedy for colic, measles, and most other infantile ailments. A cock-roach,—just think of it—when swallowed alive, was a sure remedy for hydrophobia. A man would certainly be considered *mad* at this day, to dispose of such an object in the manner described. Ants,—little red ones,—were considered to be, when swallowed alive, of great efficacy in leprosy, and as a means of strengthening the memory. Dead spiders, when taken alone, was sure cure for cancer ; if the web and dead flies therein contained where taken with it, a sovereign and effectual remedy was supposed to have been swallowed for the "King's evil."

We suppose the patients of the doctors who employed these "remedies" recoverd quite as often as do those under the care of our more learned and skillful physicians of this day. What a strange train of thought this might lead us to ! Guess we will pursue it no further.

---

Rest.—Now that the angling season has arrived, we anticipate with other true followers of Sir Isaak, to engage for a short season in the delightful and health inspiring occupation of casting the fly, for the amusement of the individual at one end of the rod, and the discomfiture, if not astonishment, of the sprightly finny beauty, likely to be ensnared by the alurements presented, at the other end.—It is, of course, understood that we purpose retaining possession of the end *first* referred to. If there is anything more than another that delights the eye and electrifies the arm of the true sportsman, it is to witness the leap of a magnificent trout as he darts from beneath some mossy bank to seize the life-like fly,—then, with a tug that fairly "takes your breath," sends your line spinning through the water to the merry rattle of the reel—whew ! the very thought of such a scene sends a thrill through our veins, and revives recollections never to be forgotten. Come, good friends, you who have joined us in many such an excursion, and who appreciate so thoroughly the delights offered by our favorite mountain streams, let us be up and doing—let's go.

